# Antimicrobial use and documented infection among hospitalized adults in South American acute care facilities during the coronavirus disease 2019 (COVID-19) pandemic

**DOI:** 10.1371/journal.pone.0343535

**Published:** 2026-02-27

**Authors:** Lauren F. Dempsey, Hanako Osuka, Olivia L. McGovern, Jose M. Munita, Maria Isabel Garzon, Icaro Boszczowski, Anne Peters, Maria Spencer-Sandino, Matias C. Salomao, Fernanda C. Lessa, Twisha S. Patel

**Affiliations:** 1 International Infection Control Branch, Division of Healthcare Quality Promotion, National Center for Emerging Zoonotic and Infectious Diseases, Centers for Disease Control and Prevention, Atlanta, Georgia; 2 Instituto de Ciencias e Innovación en Medicina, Facultad de Medicina Clínica Alemana, Universidad del Desarrollo, Santiago, Chile; 3 Servicio de Infectología, Hospital Privado de Córdoba, Cordoba, Argentina; 4 Hospital Español, Córdoba, Argentina; 5 Infection Control, Hospital das Clínicas, Universidade de São Paulo, São Paulo, Brazil; 6 Department of Infectious Diseases, Hospital das Clínicas, Universidade de São Paulo, São Paulo, Brazil; 7 Hospital Alemão Oswaldo Cruz, São Paulo, Brazil; 8 Laboratório de Investigação Médica 49 -LIM49, Faculdade de Medicina, Universidade de São Paulo, São Paulo, Brazil; Gulu University, UGANDA

## Abstract

**Background:**

Despite low bacterial and fungal infection rates, increased antimicrobial use (AU) among hospitalized patients has been reported during the Coronavirus Disease 2019 (COVID-19) pandemic. We evaluated whether COVID-19 was a driver of AU and documented bacterial or fungal infection.

**Methods:**

We conducted a retrospective cohort study in two hospitals each in Argentina, Brazil, and Chile. We included hospitalized adults with and without COVID-19 admitted during the pandemic (March 2020-February 2021) as well as a cohort admitted prior to the pandemic (March 2019-February 2020) with similar age and length of hospitalization. We performed multivariable logistic regressions to compare 1) patients with COVID-19 to those without who were admitted during the pandemic, and 2) patients without COVID-19 who were admitted during the pandemic to a similar patient population before the pandemic to characterize the association of COVID-19 or admission during the pandemic with rates of AU and infections.

**Results:**

A total of 1116 patients were included. During the pandemic, COVID-19 was not associated with receiving antimicrobials or receiving antimicrobials for a duration >48 hours, but it was associated with reduced likelihood of culture-positive bacterial or fungal infection (aOR=0.35, 95% CI: 0.19–0.64, p < 0.001). Compared to patients without COVID-19 admitted during the pandemic, patients admitted prior to the pandemic were more likely to have received antimicrobials (aOR=1.54, 95% CI: 1.15–2.07, p < 0.01), but there was no association with receiving antimicrobials for duration>48 hours or having a culture-positive bacterial or fungal infection.

**Conclusions:**

COVID-19 was not associated with an increased likelihood of AU in this cohort of hospitalized adults.

## Background

Antimicrobial resistance (AMR) is a serious global health threat, and the misuse and unnecessary overuse of antimicrobials is an important contributing factor. The Coronavirus Disease 2019 (COVID-19) pandemic exacerbated this problem, with an increase in AMR observed worldwide [1]. Global pharmaceutical sales data show that antimicrobial sales increased as COVID-19 cases increased during 2020–2022, suggesting that antimicrobials were possibly over-prescribed during the pandemic [2]. Indeed, while the magnitude varies depending on the type of antimicrobial studied, facility and patient characteristics, and timing of the pandemic, multiple countries reported increases in facility-wide consumption of antimicrobials, ranging from 5–173% [3,4]. Importantly, this increase occurred despite low rates of bacterial co-infections among COVID-19 patients (0.40–6.75%) [5,6]. In addition, unmatched pressure on healthcare facilities disrupted infection prevention and control (IPC) and antimicrobial stewardship practices due to resource shortages [7,8]. Consequently, the pandemic may have hastened the emergence and transmission of AMR [9,10]. In the United States, the Centers for Disease Control and Prevention reported that despite interrupted and slowed AMR reporting, an increase in antimicrobial-resistant infections and antibiotic use were observed during the COVID-19 pandemic [11,12].

Previously identified predictors of increased empiric antimicrobial use (AU) among hospitalized COVID-19 patients include older age, multiple comorbidities, and severity of the disease [13,14] However, data on whether COVID-19 is an independent predictor of increased AU remain limited. To evaluate what factors contributed to the increased AU during the COVID-19 pandemic and explore potential opportunities for interventions, assessments of AU among patients with and without COVID-19 admitted during the pandemic and comparisons of AU in patients admitted before and during the pandemic are needed.

Several studies conducted in South American countries reported similar findings regarding overuse of antibiotics and increased AMR [15–17]. Chile reported that both AU and the frequency of carbapenemase-producing carbapenem-resistant Enterobacterales (CP-CRE) increased after the onset of the COVID-19 pandemic [15]. Similarly, the Pan American Health Organization also reported increased CP-CRE in South and Central America during the pandemic [16]. We previously reported 6.7%−35.1% increases in rates of commonly used intravenous antimicrobials in 4 of 6 studied hospitals in Argentina, Brazil, and Chile following the onset of the pandemic [17]. At the same hospitals, we conducted a cohort study to evaluate whether COVID-19 was a driver of AU and laboratory-confirmed bacterial or fungal infections among hospitalized adults.

## Methods

### Study design and patients

We conducted a retrospective cohort study in six hospitals in South America, including two hospitals each in Chile, Argentina, and Brazil. All hospitals are acute care facilities (4 private and 2 public) that admitted COVID-19 patients at the time of data collection. We included patients admitted to adult (defined as ≥15 years of age) acute care wards from March 2019 to February 2021. Patients were excluded if they were admitted to labor and delivery wards, psychiatric wards or were diagnosed with influenza-like illness (ILI).

Patients were grouped into three cohorts based on diagnosis and admission period. In the COVID-19 cohort, patients with a COVID-19 diagnosis using laboratory results and/or discharge codes ICD-10: U07.1 and U07.2 were randomly selected if admitted between March 2020 and February 2021. Sampling of COVID-19 cases occurred proportionately across the included time period. Patients admitted between March 2020 and February 2021 without COVID-19 but with similar age and length of hospitalization (± 1 day) as those with COVID-19 were included in the non-COVID-19 cohort. Finally, patients admitted between March 2019 and February 2020, also with similar age and length of hospitalization as those with COVID-19, were included in the pre-pandemic cohort.

### Data collection

Patient characteristics abstracted from hospital medical records included demographics, comorbidities using a Modified Charlson Comorbidity Index [18], admission diagnosis, presence of sepsis, radiographic results, AU, and microbiologic information. Data were collected from June 1, 2021 through July 11, 2023 using a standardized data dictionary and entered into REDCap using a structured data collection template. All collected data were anonymized and only aggregated data are reported in the manuscript.

### Ethical approvals

This activity was reviewed by CDC, deemed not research, and was conducted consistent with applicable federal law and CDC policy (see, e.g., 45 C.F.R. part 46.102(l)(2), 21 C.F.R. part 56; 42 U.S.C. §241(d); 5 U.S.C. §552a; 44 U.S.C. §3501 et seq.). Thus, informed consent was not required. The protocol was evaluated and approved by the institutional review board at each participating healthcare facility: Hospital Alemão Oswaldo Cruz (no. 4.511.081), Hospital das Clínicas FMUSP (no. 4.689.274), Clínica Alemana-Universidad del Desarrollo (no. 2021–24), Comité Etico-científico Servicio de Salud Metropolitano Sur Oriente, Hospital Privado Universitario de Córdoba (no. 4–334), and Hospital Español.

### Study outcomes

Study outcomes were categorized into three groups: AU, bacterial or fungal infection, and clinical outcomes. AU included use of any intravenous or oral antimicrobial and was further defined as: any antimicrobials received, any antimicrobials received for a duration >48 hours, and any antimicrobials received within 48 hours of hospital admission. Antimicrobials were characterized by agent or class using the following categories: azithromycin, ceftriaxone, antibiotics used to treat community-acquired pneumonia (defined as ceftriaxone, amoxicillin/clavulanate, ampicillin/sulbactam, levofloxacin, or moxifloxacin use), anti-pseudomonal β-lactam antibiotic use (defined as aztreonam, cefepime, ceftazidime, ceftolozane/tazobactam, imipenem, meropenem, and piperacillin/tazobactam), and carbapenem use (defined as imipenem, meropenem, or ertapenem).

Bacterial and fungal infection were defined as having a positive clinical culture and further characterized by carbapenem resistance amongst gram-negative organisms. Clinical outcomes assessed included all-cause in-hospital mortality and hospital readmission within 30 days.

### Statistical analysis

Descriptive statistics were used to compare the COVID-19 cohort vs the non-COVID-19 cohort and the non-COVID-19 cohort vs the pre-pandemic cohort. We performed univariable and multivariable logistic regression analyses for three outcomes of interest to estimate odds ratios (ORs) and 95% confidence intervals (CIs): 1) receipt of any intravenous or oral antimicrobials during hospitalization, 2) receipt of antimicrobials for duration >48 hours, and 3) culture-positive bacterial or fungal infection. Although all models used the cohort of non-COVID-19 patients admitted during the pandemic as the reference group, COVID-19 and admission prior to the pandemic were tested as exposures in separate models. Based on available variables collected, the associations were adjusted for different confounders using forward stepwise regression (using P ≤ 0.05 as criteria for inclusion). ICU admission, length of hospital stay (>5 days), mechanical ventilation use, modified Charlson Comorbidity Index score (>0), oxygen therapy use, patient age > 65 years, and prior hospitalization in the past 30 days were potential confounders. All analyses were performed using SAS software, version 9.4 (SAS Institute Inc. Cary, NC) and statistical significance was defined as P ≤ 0.05 for a two-tailed test.

## Results

We included 1,116 patients (372 patients from each cohort) in the analysis. Compared to the non-COVID-19 cohort, COVID-19 patients were more likely to be admitted to the ICU (19.9% vs 15.1%), have been transferred from another acute care hospital (14.0% vs 4.3%), have symptoms or radiographic signs of pneumonia (87.9% vs. 4.6%), and require oxygen therapy (57.3% vs. 15.9%). History of a prior hospitalization and diagnosis of another type of systemic infection was less common among COVID-19 patients as compared to the non-COVID-19 cohort (8.6% vs. 16.9%, and 2.7% vs. 14.0%, respectively), as shown in Table 1. The seasonal distribution of COVID-19 and non-COVID-19 cases was similar (S1 Fig). No major differences were observed between the non-COVID-19 and the pre-pandemic cohorts (Table 1).

**Table 1:** Patient Characteristics by Cohort (COVID-19 Patients, Contemporary Non-COVID-19 Patients, Pre-Pandemic Patients) from Hospitals in Argentina, Brazil, and Chile, March 2019-February 2021.

Characteristics	COVID-19 Patients		Non-COVID-19 Patients		Pre-Pandemic Patients	
	N = 372		N = 372		N = 372	
	N	%	N	%	N	%
**Sex**						
Male	220	59.1	205	55.1	192	51.6
Female	152	40.9	167	44.9	180	48.4
**Age (years), Median [IQR]**	68 [55.5-76.0]		67.5[56.0-77.5]		66 [54.0-77.5]	
**Hospitalized in last 30 days**	32	8.6	63	16.9	61	16.4
**Modified Charlson Comorbidity Index, Median [IQR]**	1 [0, 1]		0 [0, 2]		0 [0, 1]	
**Location patient was admitted from**						
Private residence	274	73.7	319	85.8	319	85.8
Long-term care facility	11	3.0	1	0.3	3	0.8
Transferred from another acute care hospital	52	14.0	16	4.3	17	4.6
Other	29	7.8	22	5.9	20	5.4
Unknown	3	0.8	11	3.0	11	3.0
Missing	3	0.8	3	0.8	2	0.5
**Admission diagnosis of other systemic infection**	10	2.7	52	14.0	58	15.6
**Radiographic signs or symptoms of pneumonia**	327	87.9	17	4.6	17	4.6
**Sepsis or septic shock (within 48 hours of admission)**	16	4.3	10	2.7	16	4.3
**Mechanical ventilation**	23	6.2	18	4.9	14	3.8
**Non-invasive oxygen therapy**	213	57.3	59	15.9	53	14.3
**ICU admission**	74	19.9	56	15.1	63	16.9
**Length of hospital admission (Days), Median [IQR]**	6 [4 –8]		5 [3 –8]		5 [3 –8]	
**Culture collected during hospitalization**	125	33.9	121	32.8	122	33.2

*Abbreviation: IQR, Interquartile Range; ICU, Intensive Care Unit.

Table 2 shows the frequencies of patient outcomes by cohort. The proportion of patients on antimicrobials was numerically lower during the pandemic (50.0% for COVID-19 patients and 48.4% for non-COVID-19 patients) than pre-pandemic patients (58.6%). Still, pre-pandemic patients had a lower prevalence of receiving antimicrobials for longer than 48 hours (51.8% for pre-pandemic patients vs. 61.1% for COVID-19 and 60.1% for non-COVID-19 patients). More COVID-19 patients were initiated on antimicrobials within 48 hours of admission (96.2% for COVID-19 patients vs. 86.7% for non-COVID-19 patients and 91.3% for pre-pandemic patients). Median duration of AU was similar between the three cohorts (COVID-19 patients: 3.3 days, [interquartile range, 1.9 days], non-COVID-19 patients: 3.7 days [4.8 days], pre-pandemic patients: 2.9 days [2.5 days]).

**Table 2:** Clinical and Patient Outcomes by Cohort (COVID-19 Patients, Contemporary Non-COVID-19 Patients, Pre-Pandemic Patients) from Hospitals in Argentina, Brazil, and Chile, March 2019-February 2021.

Characteristics	COVID-19 Patients (n = 372)		Non-COVID-19 Patients (n = 372)		Pre-Pandemic Patients (n = 372)	
	N	%	N	%	N	%
**Any Antimicrobial Use**	185	50.0	180	48.4	218	58.6
Antimicrobial Use (duration >48 hours)	113	61.1	109	60.6	113	51.8
Average Duration of Antimicrobial Use (Days), Mean (Standard Deviation)	3.3 (1.9)		3.7 (4.8)		2.9 (2.5)	
Early Antimicrobial Use (within 48 hours of admission)	178	96.2	156	86.7	199	91.3
**Has a Culture-positive Bacterial or Fungal Infection**	18	4.8	39	10.5	37	10.0
Tested Positive for Gram-Negative Bacteria	9	50.0	31	79.5	22	59.5
Gram-Negative Bacteria is Carbapenem-Resistant	3	33.3	3	9.7	1	4.5
**In-hospital Mortality**	55	14.8	17	4.6	15	4.0
**Readmission within 30 days**	19	5.9	47	13.2	52	14.6

Culture-positive infection was less common among patients with COVID-19 (4.8%) compared to the other cohorts (non-COVID-19 patients: 10.5% and pre-pandemic patients: 10.0%), but the prevalence of carbapenem-resistance among gram-negative bacterial infections was higher in COVID-19 patients (33.3% vs. non-COVID-19 patients: 9.7% and pre-pandemic patients: 4.5%; Table 2). S1 Table details positive cultures by pathogen name and cohort. In-hospital mortality was more common among COVID-19 patients (14.8%) than for non-COVID-19 patients (4.6%) and pre-pandemic patients (4.0%), whereas hospital readmission was less common (5.9% for COVID-19 patients vs. 13.2% for non-COVID-19 patients and 14.6% for pre-pandemic patients; Table 2).

After adjusting for covariates, COVID-19 was not associated with AU in patients (aOR=0.99, 95% CI: 0.74–1.33, p = 0.97) or AU for duration >48 hours (aOR=1.17, 95% CI: 0.84–1.62, p = 0.36), but it was associated with reduced likelihood of culture-positive bacterial or fungal infection (aOR=0.35, 95% CI: 0.19–0.64, p < 0.001) compared to non-COVID-19 patients (Table 3). Covariates by final adjusted model are listed in S2-S5 Tables.

**Table 3:** Logistic Regression Results, Antimicrobial Use and Culture-Positive Bacterial Infection by Cohort (COVID-19 Patients, Contemporary Non-COVID-19 Patients, Pre-Pandemic Patients)* from Hospitals in Argentina, Brazil, and Chile, March 2019-February 2021.

Outcome	Unadjusted						Fully Adjusted*					
	Coefficient	Wald Chi-square	P-value	cOR†	Lower CI†	Upper CI	Coefficient	Wald Chi-square	P-value	aOR†	Lower CI	Upper CI
**Any Antimicrobial Use (ref: Contemporary Non-COVID-19 Patients)**												
COVID-19 Patients	0.054	0.13	0.71	1.06	0.79	1.41	-0.01	**<0.01**	0.97	0.99	0.74	1.33
Pre-Pandemic Patients	0.412	7.77	0.01	1.51	1.13	2.02	0.43	8.34	<0.01	1.54	1.15	2.07
**Antimicrobial Use Duration > 48 hours (ref: Contemporary Non-COVID-19 Patients)**												
COVID-19 Patients	0.29	3.52	0.06	1.34	0.99	1.83	0.15	0.84	0.36	1.17	0.84	1.62
Pre-Pandemic Patients	0.05	0.10	0.75	1.05	0.77	1.44	0.05	0.08	0.78	1.05	0.75	1.47
**Culture-positive Bacterial or Fungal Infection (ref: Contemporary Non-COVID-19 Patients)**												
COVID-19 Patients	-0.83	8.00	<0.01	0.43	0.24	0.77	-1.04	11.57	**<0.001**	0.35	0.19	0.64
Pre-Pandemic Patients	-0.06	0.06	0.81	0.94	0.59	1.52	-0.07	0.07	0.79	0.94	0.58	1.52

*Full list of covariates by model can be found in S2-S7 Tables.

† Abbreviation: cOR, Crude Odds Ratio; CI, 95% Confidence Interval; aOR, Adjusted Odds Ratio

Compared to non-COVID-19 patients admitted during the pandemic pre-pandemic patients were more likely to have received antimicrobials (aOR=1.54, 95% CI: 1.15–2.07, p < 0.01), but there was no association on AU for duration > 48 hours (aOR=1.05, 95% CI: 0.75–1.47, p = 0.78) or having a culture-positive bacterial or fungal infection (aOR=0.94, 95% CI: 0.58–1.52, p = 0.79). Covariates by final adjusted model are listed in S6-S7 Tables.

Comparisons of AU groups are displayed in Table 4. COVID-19 patients who were receiving antimicrobials had a higher prevalence of receiving azithromycin (46.5% vs. 4.4%), ceftriaxone (82.2% vs. 35.6%), and a community-acquired pneumonia (CAP) regimen (85.4% vs. 46.7%) compared to the non-COVID-19 cohort. Compared to COVID-19 patients, non-COVID-19 patients receiving antimicrobials were more likely to receive anti-pseudomonal β-lactam antibiotics (21.1% vs. 14.1%). Carbapenem use was similar between COVID-19 and non-COVID-19 patients (7.6% and 9.4%, respectively). Prevalence of antimicrobial agent by type was similar between pre-pandemic patients and non-COVID-19 patients.

**Table 4:** Prevalence of Antimicrobial Use by Agent Type Among Patients Receiving Antimicrobials (COVID-19 Patients, Contemporary Non-COVID-19 Patients, Pre-Pandemic Patients) from Hospitals in Argentina, Brazil, and Chile, March 2019-February 2021.

Antimicrobial Use	COVID-19 Patients Taking Antimicrobials		Non-COVID-19 Patients Taking Antimicrobials		Pre-Pandemic Patients Taking Antimicrobials	
	(N = 185)		(N = 180)		(N = 218)	
	N	%	N	%	N	%
**Azithromycin**	86	46.5	8	4.4	8	3.7
**Ceftriaxone**	152	82.2	64	35.6	75	34.4
CAP* Regimen (Ceftriaxone, Amox/Clav, Amp/Sulbactam, Levofloxacin, Moxifloxacin)	158	85.4	84	46.7	97	44.5
Anti-PsA* β-lactam Antibiotics	26	14.1	38	21.1	44	20.2
**Carbapenems**	14	7.6	17	9.4	16	7.3

* Abbreviations: CAP, Community-Acquired Pneumonia; PsA, Pseudomonas aeruginosa.

## Discussion

Our study found that COVID-19 was not an independent predictor of increased AU among patients admitted to six hospitals in Argentina, Brazil, and Chile. The rate of culture-positive bacterial or fungal infection was low among COVID-19 patients (4.8%), which is consistent with previously published reports from various countries [5]. Even though there was no significant difference in AU and AU duration between COVID-19 and non-COVID-19 patients, the rate of AU was still high (50.0% in COVID-19 patients) considering the viral etiology of the disease and the low rates of bacterial co-infection. Our findings suggest COVID-19 patients were treated for CAP despite a viral diagnosis, given the disproportionately high use of azithromycin, ceftriaxone, and other antimicrobials typically used in a CAP regimen. Such practices might highlight an opportunity for intervention by antimicrobial stewardship programs to mitigate inappropriate AU among patients diagnosed with viral infections. Although the number was small, the prevalence of carbapenem-resistance among gram-negative bacterial infections was higher in COVID-19 patients. This might suggest that IPC practices in COVID-19 wards need improvement to prevent the transmission of gram-negative bacteria. Our previous study reported limited antimicrobial stewardship and IPC activities at the same facilities during the pandemic [17]. This may also have contributed to the unnecessary use of antimicrobials among COVID-19 patients, underscoring the need for strengthened oversight and targeted interventions to optimize AU during future public health crises (e.g., pandemic).

Our previous study reported that facility-level AU increased at the onset of the COVID-19 pandemic [17]. However, in this study, AU was observed to be lower among patients admitted during the pandemic period compared to those in the pre-pandemic period. This discrepancy may be attributable to differences in the populations studied. Unlike the previous report describing facility-wide AU trends, this study focused on specific cohorts with relatively short lengths of hospitalization within a defined time frame, which may have influenced the findings. Additionally, other research has described a notable rise in AU during the early stage of the pandemic [3,19], suggesting that the timing of the study period may also play a role in the observed AU trends as our COVID-19 cohort included patients admitted from March 2020 to February 2021 as shown in S1 Fig. Another potential contributing factor is the challenges faced by facilities during the pandemic. Limited access to certain antimicrobials and the need to restrict patient admissions due to the surge in COVID-19 cases may have altered AU practices compared to the pre-pandemic period [17]. Longitudinal monitoring of AU is crucial to understand how antimicrobial prescribing practices have evolved during and after the pandemic and to draw more definitive conclusions.

Compared to previously published studies, the proportion of COVID-19 patients who received antimicrobials was lower in this study. In a study from the United States, 77.3% of COVID-19 patients were prescribed antimicrobials [20]. A large cohort study in Brazil also reported a higher rate of AU among COVID-19 patients compared to non-COVID-19 patients (70% vs 39%) [21]. The differences in rates of AU can potentially be attributed to the differences in the studied patient populations. In our study, patients with COVID-19 were relatively young and healthy, had short hospitalizations, and had less severe disease, as evidenced by low ICU admission, rates of sepsis, ventilator use, and rates of broad-spectrum anti-pseudomonal β-lactam AU.

Our study has several strengths, including being a multi-center study with patients from multiple countries. Many previous studies on facility-level AU among patients with COVID-19 were single-center. Therefore, our study provides a broader perspective on AU patterns and documented infections in South America, where such analyses have not previously been conducted. Secondly, our study design is unique in that it includes three distinct cohorts. Although AU and AMR among patients with COVID-19 have been studied, most studies lack a comparison group or do not control for disease severity. We included non-COVID-19 patients as a comparator group to determine if COVID-19 was a driver of AU. We also included a second comparison to assess how AU and documented infections were identified during the pandemic compared to a similar population of hospitalized adults admitted before the pandemic.

One of our study limitations is generalizability. The data was collected in 2020−2021, during the early pandemic period when antiviral treatment was not yet available or access was limited; access to vaccines was also restricted during this time. Further, many local and national guidelines promoted the use of antibiotics for treatment in patients with suspected COVID-19. Additionally, patients admitted to the hospital with COVID-19 during our study period may have been more likely to be treated with antimicrobials than today due to uncertainty of COVID-19 diagnosis and lack of clinical guidance on diagnosis. This may limit the applicability of our findings to the rates of AU among patients with COVID-19 in these countries currently. Another limitation is the retrospective design, which relies on hospital record review and may introduce incomplete data or misclassification. Although most COVID-19 patients continued antimicrobial treatment for more than 48 hours, we could not determine the exact indication for antimicrobial use. Additionally, the diversity of diagnoses among non-COVID patients and pre-pandemic patients makes it difficult to draw concrete conclusions regarding reasons for differences seen in rates of AU. Lastly, our study population was limited to adults (defined as ≥15 years of age). Therefore, results may not be generalizable to pediatric populations.

## Conclusions

In conclusion, our study offers valuable insights into AU patterns during the COVID-19 pandemic. Future studies should investigate the long-term impacts of the pandemic on AU and AMR and explore strategies to strengthen antimicrobial stewardship practices. Continued vigilance by antimicrobial stewardship programs is crucial to prevent the further spread of AMR, especially in the context of global health emergencies.

## Supporting information

S1 Fig Distribution of Patient Month of Hospital Admission by COVID-19 Status from Hospitals in Argentina, Brazil, and Chile, March 2020-February 2021. (DOCX)

S1 Table Frequencies of Positive Cultures by Pathogen Name and Cohort from Hospitals in Argentina, Brazil, and Chile, March 2019-February 2021. (DOCX)

S2 Table Logistic Regression Results with Covariates, Having COVID-19 (Any Antimicrobial Use, ref: Contemporary Non-COVID-19 Patients) from Hospitals in Argentina, Brazil, and Chile, March 2019-February 2021. (DOCX)

S3 Table Logistic Regression Results with Covariates, Being Pre-Pandemic Patient (Any Antimicrobial Use, ref: Contemporary Non-COVID-19 Patients) from Hospitals in Argentina, Brazil, and Chile, March 2019-February 2021. (DOCX)

S4 Table Logistic Regression Results with Covariates, Having COVID-19 (Antimicrobial Use Duration > 48 Hours, ref: Contemporary Non-COVID-19 Patients) from Hospitals in Argentina, Brazil, and Chile, March 2019-February 2021. (DOCX)

S5 Table Logistic Regression Results with Covariates, Being Pre-Pandemic Patient (Antimicrobial Use Duration > 48 Hours, ref: Contemporary Non-COVID-19 Patients) from Hospitals in Argentina, Brazil, and Chile, March 2019-February 2021. (DOCX)

S6 Table Logistic Regression Results with Covariates, Having COVID-19 (Culture-Positive Bacterial or Fungal Infection, ref: Contemporary Non-COVID-19 Patients) from Hospitals in Argentina, Brazil, and Chile, March 2019-February 2021. (DOCX)

S7 Table Logistic Regression Results with Covariates, Being Pre-Pandemic Patient (Culture-Positive Bacterial or Fungal Infection, ref: Contemporary Non-COVID-19 Patients) from Hospitals in Argentina, Brazil, and Chile, March 2019-February 2021. (DOCX)

## Abbreviations

AU: Antimicrobial use
AMR: Antimicrobial resistance
aOR: Adjusted odds ratio
CAP: Community acquired pneumonia
CDC: Centers for Disease Control and Prevention
CI: Confidence interval
cOR: Crude odds ratio
COVID-19: Coronavirus disease 2019
CP-CRE: Carbapenemase-producing carbapenem-resistant Enterobacterales
ICU: Intensive care unit
ILI: Influenza-like illness
IPC: Infection prevention and control
IQR: Interquartile range
PSA: *Pseudomonas aeruginosa*.
