# Supplementary material for: Antimicrobial use and documented infection among hospitalized adults in South American acute care facilities during the coronavirus disease 2019 (COVID-19) pandemic
Source: PLoS One. 2026 Feb 27;21(2):e0343535. doi: 10.1371/journal.pone.0343535 (PMC12948048; doi:10.1371/journal.pone.0343535)

**S1 Figure. Distribution of Patient Month of Hospital Admission by COVID-19 Status from Hospitals in Argentina, Brazil, and Chile, March 2020-February 2021**


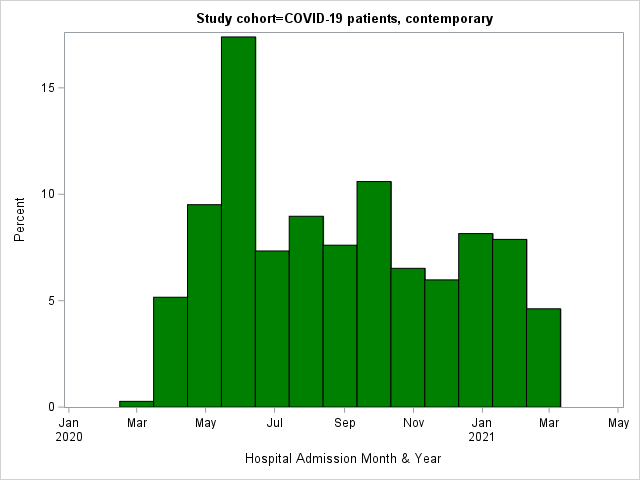

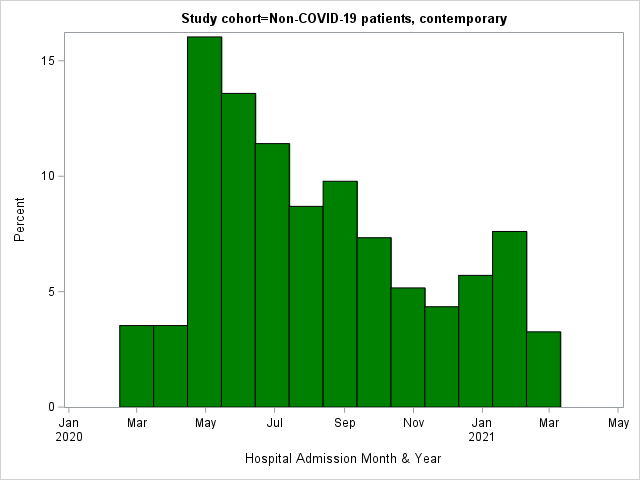

Supplement: S1 Fig — (DOCX) [file pone.0343535.s001.docx]
