# Supplementary material for: Antimicrobial use and documented infection among hospitalized adults in South American acute care facilities during the coronavirus disease 2019 (COVID-19) pandemic
Source: PLoS One. 2026 Feb 27;21(2):e0343535. doi: 10.1371/journal.pone.0343535 (PMC12948048; doi:10.1371/journal.pone.0343535)
Supplement: S2 Table — (DOCX) [file pone.0343535.s003.docx]

**S3 Table. Logistic Regression Results with Covariates, Having COVID-19 (Any Antimicrobial Use, ref: Contemporary Non-COVID-19 Patients) from Hospitals in Argentina, Brazil, and Chile, March 2019-February 2021**
